# Pupil size changes signal hippocampus-related memory functions

**DOI:** 10.1038/s41598-020-73374-9

**Published:** 2020-10-02

**Authors:** Péter Pajkossy, Ágnes Szőllősi, Mihály Racsmány

**Affiliations:** 1 Institute of Cognitive Neuroscience and Psychology, Research Centre for Natural Sciences, Magyar tudósok körútja 2, 1117 Budapest, Hungary; 2 Department of Cognitive Science, Budapest University of Technology and Economics, Egry József u. 1, 1111 Budapest, Hungary

**Keywords:** Neuroscience, Physiology, Psychology

## Abstract

A major task of episodic memory is to create unique, distinguishable representations of highly overlapping perceptual inputs. Several studies on this basic function have shown that it is based on the intact functioning of certain subregions of the hippocampus and is among the most sensitive behavioral indicators of mild cognitive impairment (MCI) and dementia. Here we assessed pupil dilation associated with performance in a widely used recognition paradigm that aims to uncover the intactness of fine-graded mnemonic discrimination. A sample of healthy undergraduate students was used. First, we showed that the correct discrimination between highly similar lure items and target items elicit larger pupil response than correct target identification. Second, we found that mnemonic discrimination is associated with larger pupil response in general as compared to target identification, regardless of whether the response was correct or not. These results suggest the pupil changes differentiate mnemonic discrimination and memory identification processes in recognition performance.

## Introduction

One of the most fascinating aspects of episodic memory is our ability to store and correctly retrieve several similar experiences in the form of unique memories (e.g. the different parking lots used near our workplace in the past year). This ability requires to build distinct representations from overlapping inputs (parking slot used yesterday vs. parking slot used today). Computational models suggest that the process of pattern separation is an efficient way to build distinct representations from similar inputs^1–3^. Pattern separation has been linked to specific subregions of the hippocampus, a neuroanatomical structure widely held to be responsible for episodic memory functions^4–6^: results of both human^7–9^ and nonhuman^10–12^ studies show that pattern separation is related to the dentate gyrus and the CA3 subregions of the hippocampus.

### Lure discrimination as behavioral indicator of pattern separation

A way to examine the behavioral consequences of pattern separation in humans is to use tasks that “characterize the behavioral outcomes of neural pattern separation,” (p 2443 in^13^), that is, tasks that tap into the distinctive character of memories. One example is the Mnemonic Similarity Task (MST, see e.g.^7,9,13,14^, for a review, see^15^), an object recognition task, in which participants have to discriminate previously seen target items from visually similar lure items. Beside targets and lures, previously not seen new items (foils) are also presented. Like in a traditional recognition memory test, participants’ task is to identify target items as old and foil items as new. Importantly, subjects are also explicitly instructed to identify lure items as similar. Such successful lure discrimination is suggested to involve the neural computation of pattern separation, because participants are required to successfully discriminate between two overlapping representations^13,16–18^. The mapping between the measures of the MST and neural computations performed by the hippocampus is supported by studies showing that successful lure discrimination in the MST engages those subregions of the hippocampus, which are assumingly associated with the process of pattern separation^7,9,16,18^. Importantly, the MST has been frequently used to prove the crucial role of pattern separation deficit in age-related decline of memory performance^13,19–21^. The results show an interesting dissociation in behavioral performance: whereas the ability of older people to recognize target items remained intact, with age they became gradually unable to successfully discriminate lure items (i.e. label them as ‘similar’). A similar dissociation between intact recognition memory performance and impaired lure discrimination emerged in psychiatric and neurological disorders as well (Schizophrenia:^22,23^ Early multiple sclerosis:^24^).

These interesting dissociations between recognition memory and successful lure discrimination reveals the importance of examining the differences between ‘similar’ responses given to lures and the correct recognition of target items. Crucially, whereas the MST is reliably linked to both hippocampal activity^7,9,16,18^ and aging deficits^13,19–21^, due to different level of explanation, the task is not intended to provide a direct measure of hippocampal pattern separation^13,15^: On the one hand, the MST provides a *behavioral* measure reflecting a *psychological* process (i.e. to label an item ‘similar’ based on the comparison of a currently presented and a retrieved item). On the other hand, pattern separation in the hippocampus is a *neural* computation acting upon representations coded by different set of neurons. Drawing inferences between concepts from different explanatory levels can be problematic (see e.g. the problem of reverse interference^25^) and thus the developers of the task suggest that the MST can be regarded as an indirect measure of hippocampal pattern separation^15^, which characterizes the behavioral outcomes of pattern separation^13^. Because of this, a more complete understanding of the selective pattern separation deficit associated with aging and other conditions requires the thorough investigation of cognitive processes underlying lure discrimination: by specifying what cognitive processes underlie lure discrimination, and how they can be linked to neural pattern separation, we can strengthen the link between the behavioral measures of lure discrimination and the neural computation of pattern separation.

One psychological mechanism which has been frequently suggested to play a role in lure discrimination is the strategy of participants to recall the original target item and then compare it with the presented lure item^7,16^. Current models of recognition memory assume that such recall-to-reject strategy is involved during the correct rejection of an item that is similar to but not the same as a studied item^26–28^. In other words, when there is an overlap between the features of a new and a studied item, it is necessary to recall the memory of the original stimulus in order to distinguish it from the present item. This process is also accompanied by a feeling of the self in the past, often termed ‘recollective experience’^29–31^. In line with this, Kim and Yassa^17^ showed that successful lure discrimination in the MST was often associated with a subjective feeling of ‘remembering’, that is participants reported a conscious recollection of details from the study episode (but see^14^).

It follows from this account that a crucial feature of labelling an item in the MST as ‘similar’ is the comparison and differentiation between two similar representations – and one can assume that pattern separation and the orthogonalization of similar representations underlies the discrimination between the two representations. Here we propose additionally, that an important step in elucidating the link between lure discrimination and the recall-to-reject strategy is the investigation of judgement veridicality. If the recall-to-reject strategy is accompanying neural pattern separation, then the mere act of lure discrimination (i.e. using the ‘similar’ response option) should be differentiated from the correctness of the judgement (i.e. the ‘similar’ response is given to a lure or not). When participants on a recognition test make the decision that the presented stimulus is not the same as the stimulus they have previously seen, the veridicality of the decision is irrelevant for lure discrimination: based on the logic of the recall-to-reject theory, participants made a "similar" decision because they retrieved a previously seen target element and considered that it did not match the stimulus being seen. For if they had not recalled the target stimulus, their decision about the recognition stimulus would be "new", and if they had recalled the target and found that it perfectly matched the stimulus they had just seen, they would have made an "old" decision. That is, the behavioral index of pattern separation in fact should be the "similar" response itself, irrespective of the status of the stimulus (whether it is a target, lure, or foil). In contrast, the correctness/veridicality of the ‘similar’ response is not about the pattern separation process itself, but about the quality of the recall process, whether participants have recalled the target element correctly and in sufficient detail.

That is, in order to understand the behavioral correlates of pattern separation, one should examine both correct and incorrect lure discrimination behavior. To this aim, one important step is to examine whether correct and incorrect ‘similar’ and ‘old’ responses can be dissociated by psychological or physiological variables accompanying the decision. Differences associated with veridicality (correct vs. incorrect response) can be associated with the quality of recall during the supposed recall-to-reject process, whereas differences between response types (‘similar’ vs. ‘old’) might be regarded as a behavioral indicator of pattern separation. Previously, pupil responses during recognition decisions has been found to be sensitive to such objective and subjective assessments of recognition memory^32–34^, thus we set out to investigate pupil size changes associated with correct and incorrect lure discrimination.

### Pupil size changes as physiological indicators of memory decisions

Task-related transient pupil dilation (PD) has been linked to several cognitive processes, including processing load of mental operations^35–38^, surprise or prediction error^39–42^, and uncertainty or response confidence^43,44^.

Importantly, pupil size changes and pattern separation processes have partly overlapping neural background. Pupil size covaries with the activity of the locus coeruleus (LC), a small brain-stem nucleus responsible for noradrenergic (NA) activity in the cortex^45,46^. The association between PD and the above described variety of cognitive functions is caused by the fact that this LC/NA system coordinates a general arousal response, which underlies the coordination of adaptive and flexible behavior in different environmental circumstances^45,47,48^. Importantly, the dentate gyrus, which is linked to neural pattern separation, receives strong direct and indirect noradrenergic projections from the LC^49,50^, and this points toward a possible involvement of the LC/NA system in the neural processes underlying pattern separation^3,51^.

Furthermore, pupillometric investigations of recognition memory revealed also interesting findings: it was found that PD is larger during correct ‘old’ responses than during correct ‘new’ responses^52,53^, an effect termed pupil old-new effect. Based on this finding, it is often suggested that PD could be used as an objective measure of memory strength (see e.g.^54^), but the evidence supporting this claim is controversial (see e.g.^55^).

One important caveat of this link is related to the finding that PD accompanying ‘old’ responses in a recognition task is found to be sensitive to both objective and subjective aspects of the decision. On the one hand, it was repeatedly shown, that incorrect ‘old’ responses to foil items are associated with increased PD, when compared to correct ‘new’ responses to foil items^32,33,54,56^, and this suggests that the subjective feeling of familiarity also triggers an increased pupil response. On the other hand, in recognition tasks, PD was found to be higher for correct than for incorrect ‘old’ decisions^33,54^ and the pupil old/new effect can be also observed when participants are instructed to pretend amnesia or respond always with ‘new’^57^. This suggest that PD can also signal prior occurrence or memory strength independently from the subjective judgement of the participants. Finally, ‘old’ responses to foils, which are semantically related to target items also show sensitivity to objective and subjective familiarity: ‘old’ responses to such lure items fell in between correct ‘old’ and correct ‘new’ responses to targets and foils, respectively^54^. Similar result was reported for lures with low degree of similarity to targets, whereas ‘old’ responses to lures with high similarity to targets were indistinguishable from ‘old’ responses to targets^58^. This pattern of results suggests that both subjective familiarity (the act of responding with ‘old’) and objective familiarity (overlap between target and lure representations) contributes to PD magnitude.

### Pupillometric investigation of the MST: the current study

**Figure 1:**
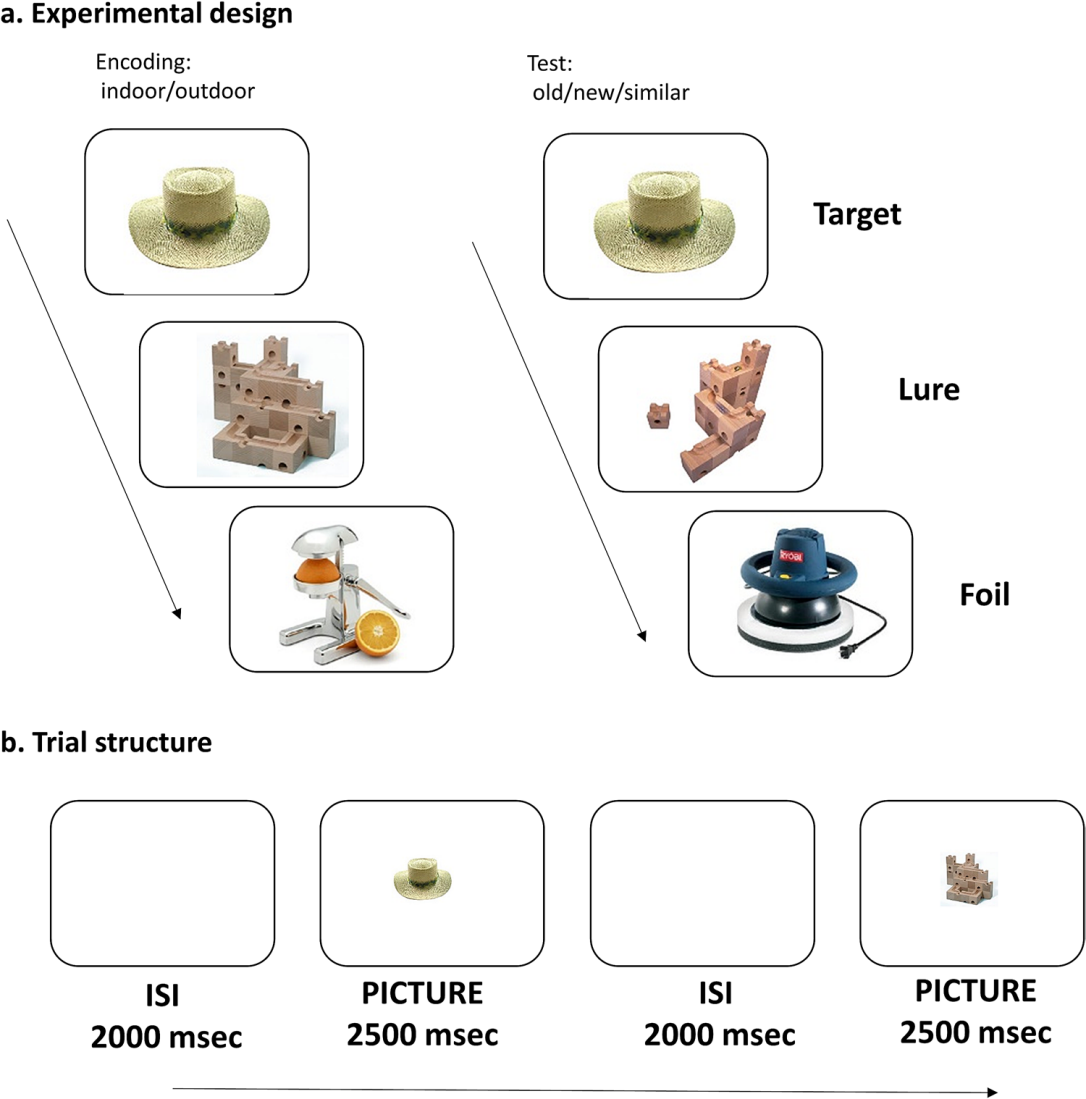
Design and Trial Structure of the Mnemonic Similarity Task. (**a**) Design: during encoding, participant made indoor/outdoor judgements about 128 pictures of everyday objects. Thereafter, in a surprise recognition test, participants have to discriminate between previously presented target pictures, similar lure items and new items. (**b**) Trial structure: Each picture was presented for 2500 ms and an intertrial interval (ISI) of 2000 ms was intermixed between pictures.

To sum up, pupil size changes and pattern separation both can be linked to the activity of the LC/NA system and PD is a sensitive measure of judgement veridicality in recognition tasks. Because of this, in the current study, we investigated the psychophysiological correlates of neural pattern separation. Specifically, we measured the size of the pupil during an MST. The design of the task is depicted on Fig. 1a-b. In the encoding phase, healthy undergraduate participants (N = 22, 14 woman, M_age_ = 23.1 years, SD_age_ = 1.9) were presented with 128 pictures of everyday objects and were asked to judge whether they are used indoor or outdoor. This was followed by a surprise recognition test, where participants were presented 64 target items (pictures seen in the encoding phase), 64 lure items (pictures, which were visually similar to, but not identical with one of the pictures seen in the encoding phase), and 64 foil items (previously unseen new pictures). Participants were asked to respond to these item types with ‘old’, ‘similar’, or ‘new’, respectively. Item presentation and information processing associated with the response triggered a dilation of the pupil, which was measured using an infrared video-based eye-tracker.

One participant was excluded due to poor data quality, thus data of 21 participants were analyzed. Furthermore, in some auxiliary analyses, due to insufficient observations (fewer than four observations in a condition), for some of the participants and for some of the conditions, pupil data could not be meaningfully interpreted. Because of this, these analyses were conducted after excluding these participants (see details in “Methods”).

To enable comparison with previous pupillometric investigations of recognition memory, we first investigated pupil responses associated with correct responses to targets, lures and foils (i.e. ‘old’ responses to targets vs. ‘similar’ responses to lures vs. ‘new’ responses to foils). On the one hand, we attempted to replicate the pupil/old new effect also in the context of mnemonic discrimination (i.e. when target items are presented together with lures and foils): we predicted that target hits evoke larger PDs than foil correct rejections. On the other hand, we predicted that successful lure discrimination will be associated with larger PDs than target hits: the recall-to-reject process presumably requires more processing resources, which causes the pupils to dilate more. The association between recollection of sensory details and the recall-to-reject strategy also points toward larger PDs associated with correct lure discrimination, because recollection is associated with large PDs (see e.g.^32,54,59^).

This analysis, however, confounds the effects of item type and response type and does not account for incorrect responses. Thus, we also investigated whether response type (‘old’ / ’similar’ / ’new’), judgement veridicality (correct / incorrect) or both can be associated with distinctive pupil responses. We had two specific hypotheses: First, we predicted that ‘similar’ responses should be associated with larger PDs, as compared to ‘old’ responses, irrespective of judgement veridicality. Specifically, if the ‘similar’ response and the underlying recall-to-reject process are correlates of pattern separation, then it should show the same psychophysiological signature regardless of whether the result of the process is correct or not. Second, based on previous research^33,54^, we predicted that PDs for incorrect ‘old’ responses will be in between PDs measured for correct ‘old’ and correct ‘foil’ items, respectively.

Finally, two control analyses were also conducted. Due to the intimate relationship between the measures of reaction time (RT) and pupil size (see e.g.^60,61^), and previous findings showing RT differences in the MST^16^, to control for possible confounding effects, we also examined RT differences between conditions. Besides, pupil size is sensitive to changes in stimulus luminance^62–64^, thus we also conducted control analyses to rule out that differences in stimulus luminance have affected our results.

## Results

### MST performance

The distribution of the three response types (‘old’, ‘new’, and ‘similar’) for the different item types (target, lure, and foil) are presented in Table 1. Whereas targets and foils were identified with high accuracy as old and new, respectively, on average, 48% of the lures were identified as similar, and 37% of lure items was labelled old. This pattern is similar to the performance reported in other samples of healthy university students (see e.g.^13,17^).

**Table 1:** Distribution of responses in the MST.

		Item type		
		Target	Lure	Foil
Proportion of responses (%)	'old'	0.76 (0.03)	0.37 (0.03)	0.02 (0.01)
	'similar'	0.15 (0.02)	0.48 (0.03)	0.1 (0.01)
	'new'	0.06 (0.01)	0.12 (0.02)	0.84 (0.01)

Mean proportion of ‘old’, ‘similar’ and ‘new’ responses for target, lure and foil items (standard deviation in parenthesis).

### Reaction time differences between conditions

**Figure 2:**
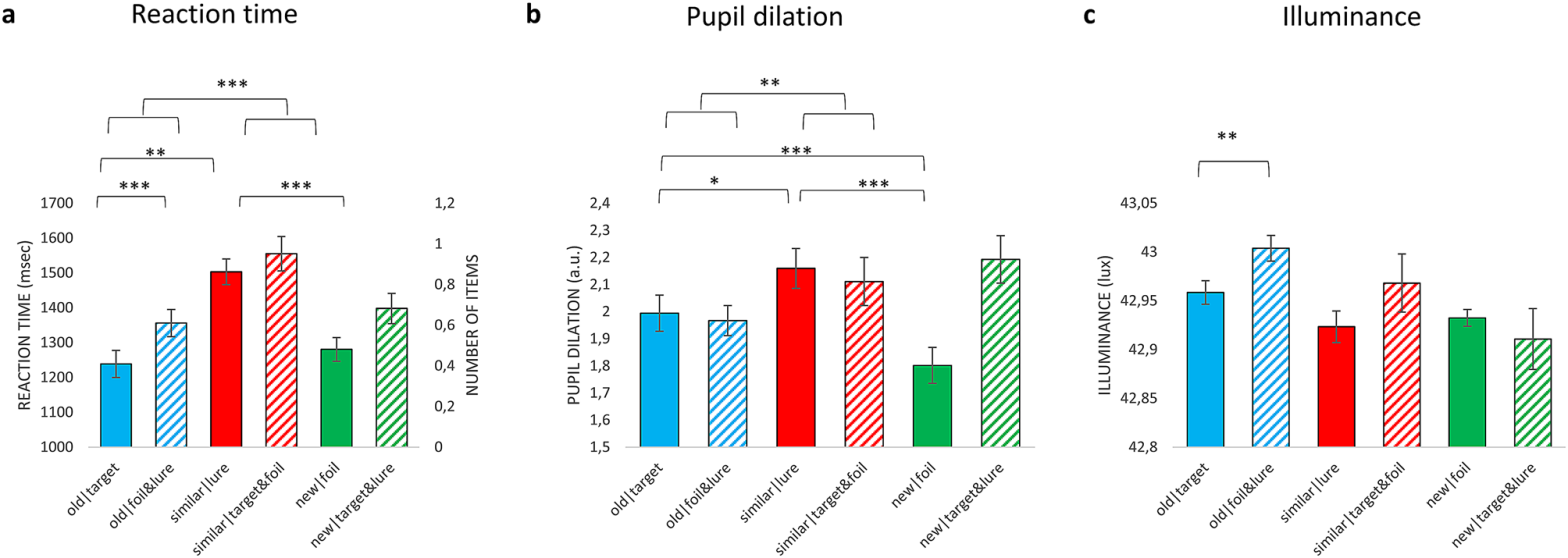
Differences between the experimental conditions in reaction time (**a**), pupil dilation (**b**) and illuminance (**c**). (**a**) Mean reaction time for correct and incorrect ‘old’, ‘similar’ and ‘new’ responses. (**b**) Mean pupil dilation for correct and incorrect ‘old’, ‘similar’ and ‘new’ responses. (**c**) Mean illuminance measured during correct and incorrect ‘old’, ‘similar’ and ‘new’ responses. Significance is indicated only for comparisons which were reported in the results section. Due to participant exclusion, the bar representing incorrect ‘new’ responses to foils represents data from 18 participants, whereas the other bars represent data from all participants. Because of this, significance between the difference for correct and incorrect ‘new’ responses are not indicated with asterisk on the graph. *p < .05; **p < .01; ***p < .001; Error bars represent the standard error of the mean.

The mean RTs for the different condition of interest are presented in Fig. 2a. The average number of trials in each condition is also presented with dashed line. As we aimed to investigate, whether differences in RT and PD mirror each other, the same analyses were conducted, as planned for PDs.

First, we examined RTs of correct responses using a one-way repeated measures ANOVA with response type (‘old’ vs. ‘new’ vs. ‘similar’) as an independent factor. The main effect was significant, F(2,40) = 26.32, *p* < 0.001, ηp^2^ = 0.57. Pairwise comparisons revealed that correct ‘similar’ responses to lures (similar|lure) were slower, as compared to both correct ‘old’ responses to targets (old|target), F(1,20) = 42.22, *p* < 0.001, ηp^2^ = 0.68, and to correct ‘new’ responses to foils (new|foil), F(1,20) = 36.81, *p* < 0.001, ηp^2^ = 0.65. The mean RT of correct ‘old’ and ‘new’ responses did not differ, F(1,20) = 1.08, *p* = 0.31, ηp^2^ = 0.05.

Second, to examine the effect of judgement veridicality, a two-way repeated measures ANOVA with response type (‘old’ vs. ‘similar’) and judgement veridicality (‘correct’ vs. ‘incorrect’) was conducted: we found a significant main effect of both response type, F(1,20) = 30.29, *p* < 0.001, ηp^2^ = 0.60, and judgement veridicality, F(1,20) = 10.14, *p* = 0.005, ηp^2^ = 0.34, and no significant interaction of the factors, F(1,20) = 2.66, *p* = 0.12, ηp^2^ = 0.12. Finally, we compared RTs of correct and incorrect ‘old’ responses, and found that the latter were significantly slower, t(20) = 4.64, *p* < 0.001, d = 0.95.

### Pupil dilation associated response type and judgement veridicality – time course analysis

**Figure 3:**
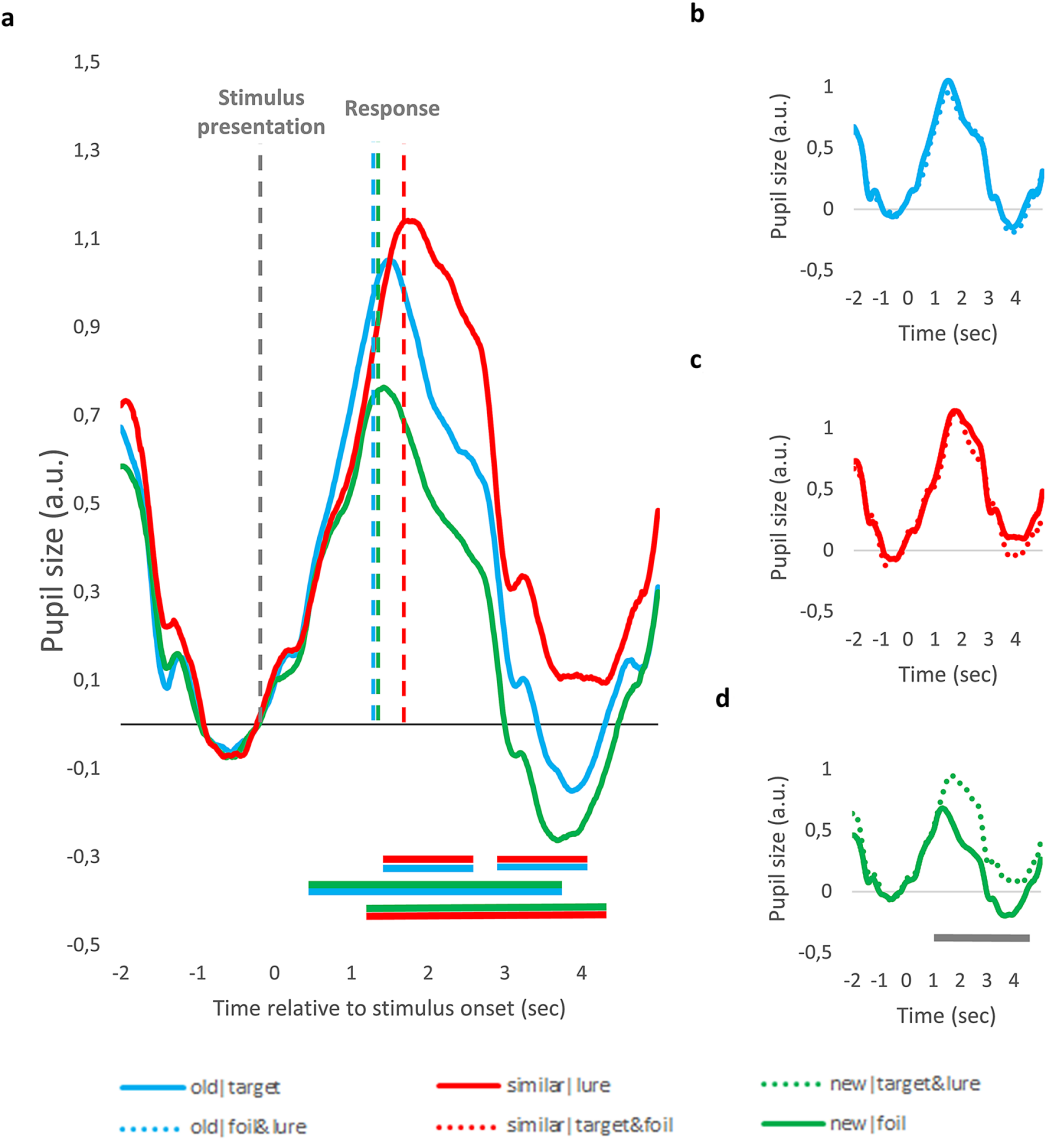
Stimulus-aligned Pupil Size Changes for the Different MST Conditions. (**a**) Pupil size changes evoked by correct ‘old’, ‘similar’ and ‘new’ responses. (**b**–**d**) Pupil size changes depicted separately for correct and incorrect ‘old’ (**b**), ‘similar’ (**c**) and ‘new’ (**d**) responses. Data are aligned to stimulus onset. Values are standardized and baseline corrected: the mean of the 500 ms preceding stimulus onset is subtracted from each data point. Time is shown relative to the onset of the trial. Horizontal lines below the pupil size curves indicate significant difference between conditions in that time-window (*p* < .05 for at least 500 ms, see text for more detail). The color of the line refers to the two conditions being compared, e.g. blue-red line indicates significant difference between ‘old’ and ‘similar’ response condition. Due to participant exclusion, Panel (**d**) represents data of 18 participants, whereas the other graphs represent data from all participants.

To investigate whether there are differences in the time course of pupil size changes between the conditions, we segmented raw data into separate data samples of seven seconds representing each trial (from two seconds before until five seconds after the stimulus presentation). Raw pupil size values were first standardized and then baseline corrected by subtracting from all data points the mean pupil size of the 500 ms preceding stimulus presentation. As can be seen on Fig. 3, there are marked differences between pupil size changes associated with the different conditions.

To test the significance of these differences, for each time-point, we used participant-level data samples, and compared pupil size values of different conditions using paired samples t-tests. To exclude false alarms due to the high number of comparisons, significant differences between two curves were only interpreted if the *p* value of these t-tests was below 0.05 for a consecutive period of at least 500 ms. The periods with significant differences extending over 500 ms are marked with horizontal lines in Fig. 3 below the pupil curves. As can be seen on Fig. 3a, the similar|lure condition was associated with larger pupil response, as compared to the old|target condition, whereas the new|foil condition was linked to the smallest PD. Differences between pupil responses associated with correct and incorrect responses are presented on Fig. 3b-d. Pupil responses were larger for incorrect ‘new’ responses to targets and lures (new|target&lure), as compared to correct ‘new’ responses to foils. In contrast, there was no difference between pupil responses evoked by correct ‘similar’ responses given to lures and incorrect ‘similar’ responses given to targets or foils (similar|target&foil). Similarly, PD did not differ for correct ‘old’ responses given to targets and incorrect ‘old’ responses given to lures and foils (old|lure&foil).

**Figure 4:**
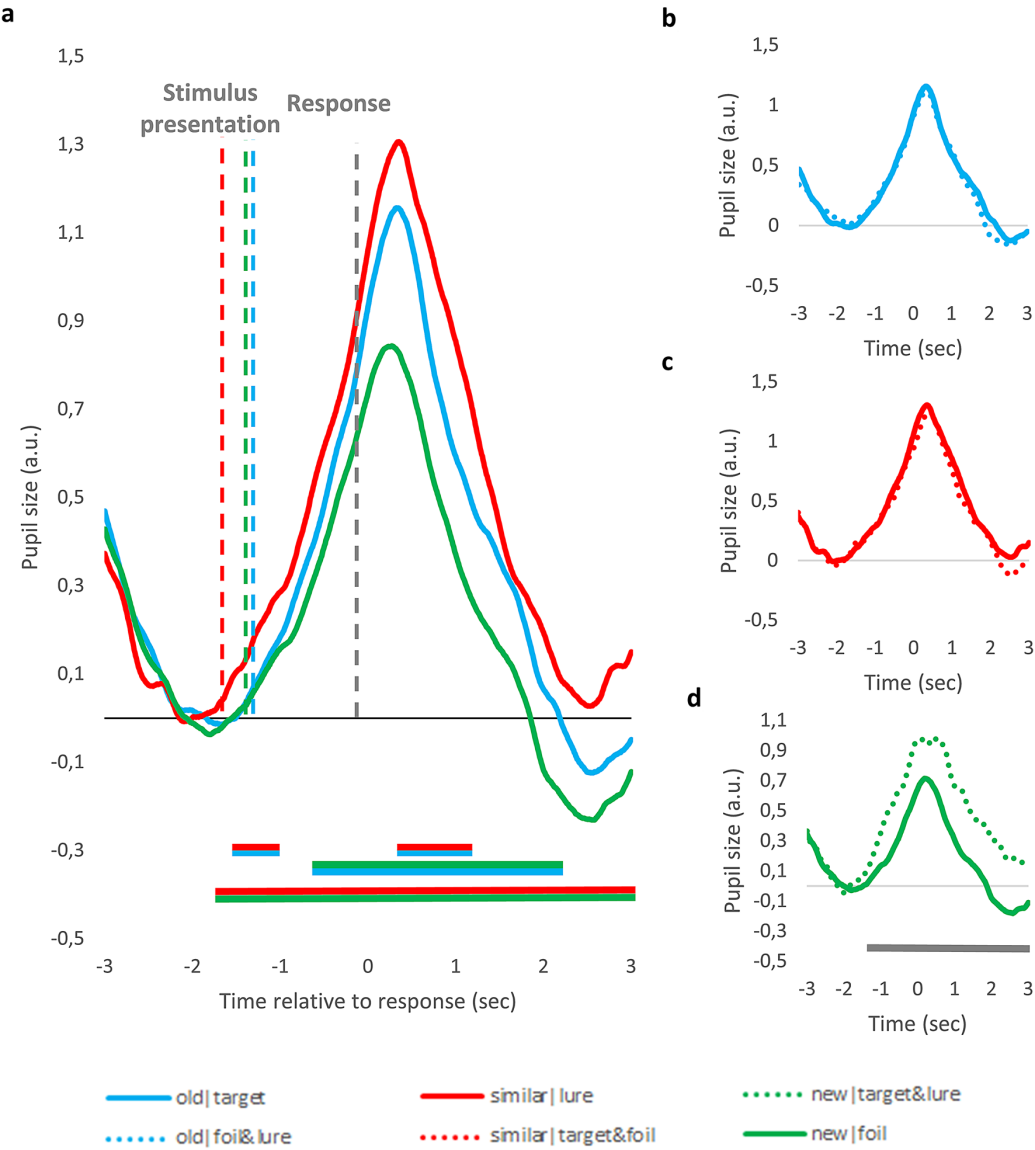
Response-aligned Pupil Size Changes for the Different MST Conditions. (**a**) Pupil size changes evoked by correct ‘old’, ‘similar’ and ‘new’ responses. (**b**–**d**) Pupil size changes depicted separately for correct and incorrect ‘old’ (**b**), ‘similar’ (**c**) and ‘new’ (**d**) responses. Data are aligned to the behavioral response. Values are standardized and baseline corrected: the mean of the 500 ms preceding stimulus onset is subtracted from each data point. Time is shown relative to the behavioral response. Horizontal lines below the pupil size curves indicate significant difference between conditions in that time-window (*p* < .05 for at least 500 ms, see text for more detail). The color of the line refers to the two conditions being compared, e.g. blue-red line indicates significant difference between ‘old’ and ‘similar’ response condition. Due to participant exclusion, Panel (**d**) represents data of 18 participants, whereas the other graphs represent data from all participants.

Importantly, there were significant RT differences between the conditions (see previous section and also vertical lines depicting average RT on Fig. 3a). Because motor responses also evoke pupil dilation^65,66^, the different timing of these motor responses might have also contributed to the demonstrated effects. To rule out this possibility, the above described analyses were repeated, but this time the raw data were segmented into response-aligned segments: for each trial, the three seconds before and after the response was selected. As can be seen in Fig. 4, the pattern of results remains unchanged – with this analysis, motor-related pupil responses are aligned in time, thus their different timing cannot contribute to the differences between conditions.

### Pupil dilation associated response type and judgement veridicality – peak pupil dilation

To conduct more complex, multivariate statistical analyses, which specifically targets our hypotheses, we quantified the magnitude of pupil dilation for each trial by computing peak dilation, that is the maximal dilation of the pupil relative to the baseline. Due to its robustness to confounds related to RT differences, we used response-aligned data to compare peak PD for the different conditions (using stimulus-aligned data leads to similar pattern of results, see details in supplementary material). The mean peak PD values are presented in Fig. 2b.

First, we tested PD associated with correct responses, using a one-way repeated measures ANOVA with correct response type, as an independent factor (old|target vs. similar|lure vs. new|foil). The main effect was significant, F(2,40) = 17.07, *p* < 0.001, η_p_^2^ = 0.46. To further explore differences between the conditions, simple contrasts were used, which showed that correct ‘similar’ responses trigger larger PD than both correct ‘old’ responses, F(1,20) = 7.19, *p* = 0.014, η_p_^2^ = 0.26, and correct ‘new’ responses, F(1,20) = 26.74, *p* < 0.001, η_p_^2^ = 0.57. We also found a significant pupil old-new effect, as PD accompanying correct ‘old’ responses was higher than PD associated with correct ‘new’ responses, F(1,20) = 13.80, *p* = 0.001, η_p_^2^ = 0.41. That is, correct responses in the MST were associated with marked differences in evoked pupil responses.

Second, we tested whether the difference between ‘similar’ and ‘old’ responses is dependent on judgement veridicality. To this aim, we used a two-way repeated measures ANOVA with judgement veridicality (correct vs. incorrect) and response type (‘old’ vs. ‘similar’), as within-subject factors. The results showed that response type had a significant main effect, F(1,20) = 12.37, *p* = 0.002, η_p_^2^ = 0.38, whereas no significant effect was detected either for judgement veridicality, F(1,20) = 0.71, *p* = 0.41, η_p_^2^ = 0.04, or for the interaction of the factors, F(1,20) = 0.05, *p* = 0.83, η_p_^2^ = 0.01. That is, ‘similar’ responses evoked larger pupil responses than ‘old’ responses, irrespective of judgement veridicality.

Importantly, in this analysis, we merged responses given to different item types in the incorrect response conditions (e.g. incorrect ‘old’ responses could be given to both lures and foils). Responses to different item types might involve different processes, thus we repeated our analysis by investigating only ‘old’ and ‘similar’ responses given to targets and lures (combinations of incorrect responses involving either foils or ‘new’ responses were rare, see Table 1). The main effect of response type (‘old’ vs. ‘similar’) was significant, F(1,17) = 13.93, *p* = 0.002, η_p_^2^ = 0.45, whereas no significant effect was detected for item type (target vs. lure), F(1,17) = 0.37, *p* = 0.55, η_p_^2^ = 0.02, and no significant interaction emerged, F(1,17) = 0.27, *p* = 0.61, η_p_^2^ = 0.02.

Third, we tested whether judgement veridicality affects PDs associated with ‘old’ responses, using paired samples t-test. In line with the time course analysis (see Fig. 3b and 4b), we found no significant differences between the old|target and the old|foil&lure conditions, t(20) = 0.44, *p* = 0.63, d = 0.07. As ‘old’ responses to foils were rare, this difference also suggests that ‘old’ responses to target and foils do not differ (this was also indicated by the previous analysis).

Fourth, although not a priori predicted, we also investigated differences between pupil response between correct and incorrect ‘new’ responses, because the time course analysis suggested significant PD differences. We used paired samples t-test, and found that peak PD is significantly higher for incorrect ‘new’ responses to targets and lures, as compared to correct ‘new’ responses to foils, t(17) = 4.84, *p* < 0.001, d = 1.13. Due to the low number of observations, we could not disentangle incorrect ‘new’ responses to targets and lures, respectively.

To sum up, we found that PDs to correct ‘similar’ responses to lures were larger than correct ‘old’ responses to targets, and both were higher than correct ‘new’ responses to foils. Furthermore, ‘similar’ responses evoked larger PDs than ‘old’ responses irrespective of judgement veridicality and item type. PDs for correct and incorrect ‘old’ responses did not differ, but incorrect ‘new’ responses were associated with larger PDs, as compared to correct ‘new’ responses.

### Control of luminance and illuminance

Pupil size is sensitive to changes in stimulus luminance^62–64^, thus the experimental design was constructed in a way to ensure that the demonstrated PD differences are not confounded by brightness differences between stimuli (e.g. randomization of stimuli, see “Methods” for a detailed description). Nevertheless, assignment of stimuli to correct and incorrect response conditions depends on participant’s responses, and so cannot be a priori randomized.

To control for this issue, we measured the amount of incoming light on participant’s eye during the examination of different pictures, that is the level of illuminance (we used illuminance instead of stimulus luminance due to the specifics of our experimental setting, see “Methods” for details). Illuminance measured during the presentation of a picture significantly and negatively predicted the average pupil response (pooled across the different conditions), as revealed using linear regression analysis (model parameter: R^2^ = 0.13, F [1,383] = 59.97, *p* < 0.001; predictor parameters: b = −0.359, SD(b) = 0.04, β = −0.368, see Supplementary Fig. S1 for a scatterplot).

To check whether this link between illuminance and PD influences our results, we calculated for each participant the average illuminance during the pictures presented in different conditions (see Fig. 2c for mean illuminance associated with the different conditions). We conducted all the analyses reported in previous section also with the illuminance values, to check whether there are significant differences between relevant comparisons. We found that incorrect responses were associated with higher illuminance level, F(1,20) = 4.31, *p* = 0.051, η_p_^2^ = 0.18, which was due to the fact that pictures presented during correct ‘old’ responses were associated with higher illuminance, as compared to incorrect ‘old’ responses, t(20) = 3.07, *p* = 0.006, d = 57. This might be due to the fact that highly similar lures were associated with somewhat higher illuminance values (see details in supplementary material). No other main effect, interaction or planned contrast was significant (all Fs < 0.2.81, all ts < 1.28. all ps > 0.11, see supplementary material for details).

Importantly, the differences between conditions are in no cases more than 0.05 lx. The results of the linear regression analysis reveal that one lux increase in illuminance is associated with 0.386 standardized unit decrease in PD (i.e. the value of the unstandardized regression coefficient). Thus, the illuminance differences between conditions can cause only small changes in PD values (around 0.01–0.03 standardized unit), and this is one order of magnitude below the PD differences found between the conditions (0.1–0.3 standardized units).

Nevertheless, to rule out any possible confounding effect of illuminance, we made an additional analysis using a linear mixed modelling. This statistical method allows to investigate the effect of interest (i.e. fixed effects, differences between conditions) and at the same time enables to control for the effects resulting from differences between certain features of stimuli on a trial-by-trial level. Using this method, we showed that after accounting for the effect of illuminance, there was still a significant difference between PDs of correct and incorrect ‘new’ responses, χ^2^ (1) = 13.97, *p* < 0.001, whereas for the other two response types, no significant difference between PDs to correct and incorrect responses were found (‘old’ responses: χ^2^ (1) = 0.09, *p* = 0.75; ‘similar’ responses: χ^2^(1) = 0.41, *p* = 0.51). Thus, we replicated the results of the ANOVA analysis with the statistical control of illuminance differences between correct and incorrect trials.

To sum up, pictures presented on incorrect trials tended to be somewhat brighter, but the differences are small and do not explain PD differences between the conditions – this is also supported by the results of the linear mixed model analysis, where illuminance differences between pictures are controlled statistically.

## Discussion

In this study, we examined pupil dilation differences in a task assessing mnemonic discrimination between highly similar photographs of everyday objects. First, we demonstrated that correct ‘similar’ responses given to the lures (i.e. lure discrimination) elicit larger PD than correct ‘old’ responses given to the targets (i.e. recognition of target items). Second, we also showed that this difference is not related to veridicality: ‘similar’ responses in general were associated with larger PD – and it did not depend on whether the response was correct or not. Third, we replicated the pupil old-new effect (see e.g.^52,54,57^) in the context of the MST: target hits were associated with larger pupil dilation, as compared to the correct rejection of foils. Interestingly, RT differences between conditions mirrored PD differences between correct ‘similar’ and ‘old’ responses, but they diverged when correct ‘old’ and ‘new’ responses were compared. In the following, we will discuss these results in more detail.

### Enhanced pupil dilation associated with the feeling of similarity

One intriguing aspect of our results is that pupil responses triggered by ‘similar’ and ‘old’ responses were not sensitive to judgement veridicality: correct and incorrect ‘similar’ responses were associated with similar PD, and both evoked larger pupil responses than correct or incorrect ‘old’ responses. Importantly, ‘similar’ responses were also significantly slower than ‘old’ responses. This pattern of findings suggests, that the mere feeling of similarity (i.e. labelling an item similar and discriminating it from a previously seen object) exhibits a distinctive psychophysiological signature, as compared to the feeling of recognizing an item as old. An important further question concerns what is the cause for this distinct arousal response.

First, it might be the case that enhanced PD is a sign of the recall-to-reject strategy: the comparison of an on-screen stimulus to a memory representation requires working-memory processes and so increases processing load. Because the magnitude of PD correlates with processing load^35–38^, the increased PD accompanying the ‘similar’ response might be caused by increased processing load of the recall-to-reject process. Furthermore, because recollective processes are also thought to be associated with increased PD^32,54,59^, our results are also in line with the suggested involvement of recollection in the recall-to-reject strategy. Finally, increased RTs are associated both with cognitive control processes^67–69^ and with recollection^70^, thus our results are in line with the assumption, that the ‘similar’ response is accompanied by increased processing load and recollection.

Second, increased PDs might signal differences in decision variables associated with labeling an item ‘similar’ vs. ‘old’. The LC/NA system and the evoked PDs have been repeatedly associated with surprise^39,41^ and mismatch detection^71,72^, thus evoked PDs might signal that a mismatch was detected between the stored memory representation and the presented input. Alternatively, our results might signal reduced response confidence and uncertainty. In a recent study^14^, it was found that successful lure discrimination relies less on recollection, and responses are more frequently based on a sense of familiarity without conscious recollection of the target item. It was also shown that subjects are less confident in their correct ‘similar’ responses, as compared to their correct ‘old’ responses. Because low response confidence was shown to be accompanied by both large RTs^73,74^ and large PDs^43,44^, our results are also compatible with the assumption proposing that ‘similar’ responses are associated with reduced response confidence and familiarity^14^.

These two explanations rest on somewhat contradictory assumptions: One the one hand, according to the recall-to-reject account, ‘similar’ responses are associated with increased cognitive load and vivid recollection of the original target item. On the other hand, it might be the case that ‘similar’ responses reflect a vague feeling of familiarity and decision uncertainty. In line with our results, both explanations would predict increased PDs and RTs. Importantly, these accounts are not mutually exclusive: It might be also the case that ‘similar’ responses sometimes involve recollection and recall-to-reject, whereas sometimes are a result of uncertainty and familiarity. Interestingly, ‘similar’ responses were linked to both familiarity and recollection in the two studies investigating this issue^14,17^, only the relative frequency with which these processes were associated with the ‘similar’ response differed. Thus task-variables (instruction, task difficulty) might influence whether the ‘similar’ response rather reflects recall-to-reject processing or is more driven by uncertainty. Importantly, only the recall-to-reject account provides a clear link between neural pattern separation and lure discrimination in the MST: the comparison and discrimination of similar representations might require the pattern separation process conducted by the hippocampus. In contrast, if ‘similar’ responses are driven by a feeling of familiarity and decision uncertainty, then the involvement of pattern separation is less straightforward. Because of this, further research of this issue is warranted to establish under what circumstances can the ‘similar’ response be taken as a behavioral proxy of pattern separation.

Finally, a third, direct link between neural pattern separation and the pattern of PD differences can be also proposed: previously it was suggested that NA projections from the LC to the hippocampus play a role in neural pattern separation^3,51^, thus increased PD associated with the ‘similar’ response might be taken as a sign of increased noradrenergic activity associated with pattern separation. Importantly, this explanation requires further validation, as it suggest a direct link between neural and psychological phenomena, and so is subject to the problems of inference detailed in the introduction.

Further research is required to clarify the contribution of the above factors in the elevated pupil response associated with the feeling of similarity. Irrespective of the exact cause, this pattern of results implies that the behavioral correlate of neural pattern separation is the “similar” response itself – regardless of the correctness of the decision.

### Pupil responses associated with objective and subjective factors in recognition memory

We replicated the pupil old-new effect in the context of the MST. This finding is noteworthy, because the MST differs from a traditional recognition memory test with respect to both the presented stimuli and the available response options. The fact that these differences leave the pupil-old new effect unaffected provides further evidence that this effect accompanies the access of a memory representation.

Interestingly, whereas the PD differences between correct ‘similar’ and ‘old’ responses were accompanied by significant differences in RT as well, this was not the case for the comparison of correct ‘old’ and ‘new’ items. Here, a marked difference in PD was linked to no changes in RT. This result also supports the suggestion, that the pupil old/new effect cannot be linked to cognitive load or decision uncertainty, but is associated with memory strength.

Similar to previous results, we could also differentiate between objective and subjective factors affecting the link between memory processes and pupil responses, but the pattern of our results somewhat diverge from those published in previous studies.

First, in previous studies, it was shown that false recognition was usually associated with smaller PD than correct recognition^33,54,56^, which can be taken as an evidence that PD triggered by ‘old’ responses are not only mediated by decision-related variables, but also by the strength of the underlying memory trace. In contrast to this, we could not demonstrate any difference between PD triggered by correct and incorrect ‘old’ responses. This might be explained by the fact that in the above mentioned studies incorrect ‘old’ responses to foils were investigated whereas such responses in our study were extremely rare (on average only 2% of all trials). In our data set, incorrect ‘old’ responses were almost exclusively given to lure items. Thus, it is possible, that in some of the incorrect ‘old’ responses participants were engaged in a recall-to-reject processing with the result of incorrectly labelling a lure item as ‘old’. In such cases, this process might have also contributed to PD and might so overshadow differences caused by memory strength.

Second, item type exerted a large influence on PD triggered by ‘new’ responses: incorrect ‘new’ responses to targets and lures elicited significantly higher PD than correct rejection of foils. This is an intriguing finding as it suggests that PD might signal prior encounter with a stimulus also in the absence of conscious recognition of that item. Interestingly, whereas nominal differences between PD evoked by misses and correct rejections have been reported previously^33,75^, to the best of our knowledge, this is the first study reporting significant differences. Further research has to clarify whether this difference can be also demonstrated in standard recognition paradigms or is caused by the specifics of the MST design.

To sum up, in line with previous research, we also found that PD associated with recognition memory can signal memory strength or prior encounter with a stimulus, and it can be also related to psychophysiological signature of the decision processes involved in a recognition. Interestingly, the pattern of results using the MST differs from those reported for simple recognition memory tasks, which highlights the important role of task-specific details (e.g. presentation of lure items or the availability of a ‘similar’ response option) in determining the influence of such objective and subjective factors on PD.

### Implications for the clinical use of the MST

Our results clearly suggest that lure discrimination and recognition memory can be discriminated by the underlying psychophysiological processes. This might be an important information for clinical application of the MST: whereas previous research proved that the MST assesses behavioral output associated with neural pattern separation^7,9^, our data open up the possibility that performance on the task might be influenced by other processes as well. For example, our data showing increased PDs are compatible with the suggestion that successful lure discrimination is associated with increased cognitive load associated with the recall-to-reject strategy. If this assumption were true than group differences in MST performance could be also caused by differences in how participants are able to exert controlled attention during an effortful task (see e.g.^76^). Importantly, Stark, Stevenson, Wu, Rutledge, and Stark^20^ ruled out this interpretation in the case of aging deficits, by using a simple old-new test format which is less reliant on the recall-to-reject strategy. But with the increasing use of the standard MST test format to test hippocampal dysfunction in different psychiatric and neurological conditions (for a review, see^15^), we suggest that tight control of such confounding factors is required to reliably assess behavioral proxies of hippocampal function.

### Limitations

One important limitation of our research must be considered. For the sake of comparability with other studies using the MST, we used the original, colored pictorial stimuli, which differ in luminance level and color. Because pupil size is affected by the color and luminance changes of the focally presented stimuli^62–64^, this is a potential confound of our research. We made several measures to avoid that luminance differences between MST pictures confound our results. By using a white background before the presentation of the darker stimuli, we restricted any luminance related pupil size changes to the darkness reflex, and thus excluded any confounding effect from the different time course associated with the darkness and the lightness reflex^64^. Furthermore, we randomized the MST pictures between conditions and so the average darkness reflex evoked during the presentation of lures, targets and foils was equated in our sample. Finally, we measured average illuminance during presenting the different MST pictures and found no significant differences in illuminance levels associated with the different conditions, except the difference between correct and incorrect responses. Importantly, the magnitude of differences in illuminance predicts only small changes in PD, which are one order of magnitude smaller than PD differences between conditions. Finally, we conducted linear mixed model analyses, which enable to statistically control for the effect of illuminance on a trial level: these analyses also suggested that illuminance differences do not confound our results.

Thus, whereas some caution is required in interpreting the differences between correct and incorrect responses, we suggest that differences in stimulus luminance cannot explain the overall pattern of our results.

## Conclusion

In this study we have shown that lure discrimination (i.e. labelling lure items as similar) evokes larger pupil responses, as compared to recognition memory (i.e. labelling a target item as old). Whereas previous studies highlighted neurobiological differences, our results provide evidence that these two functions can be also discriminated by a basic psychophysiological variable. Importantly, the difference was related to the response itself, and was unrelated to judgement veridicality. That is, the feeling of similarity evokes larger pupil response, as compared to the feeling of recognizing an item – regardless of the correctness of this feeling. We propose that the similar response itself can be taken as a behavioral correlate of the pattern separation process.

## Methods

### Participants

Twenty-two participants participated in our study for partial credit in introductory psychology courses (14 woman, M_age_ = 23.1, SD_age_ = 1.9). They all gave informed consent. One participant was excluded from data analysis due to poor data quality. The experiment was conducted in accordance with the relevant guidelines and regulations for research involving human subjects, and it was approved by the United Ethical Review Committee for Research in Psychology, Hungary.

### Procedure

The experiment was conducted in a dimly lit room. Pupil size was measured using an SMI RED HiSpeed tower-mounted eye-tracker. Participants were seated before a standard monitor and were asked to place their head into the chinrest of the eye-tracker.

### Stimuli

We used the stimulus set developed by Stark, Yassa, Lacy and Stark^13^. This set contains pairs of pictures of everyday objects, which are similar to each other to a varying degree, that is they are each other’s lures (see Fig. 1a). The degree of similarity between such lure pairs was quantified in a study in which participants were asked to discriminate between previously seen target items and lures^13^. They categorized the lure pairs into five distinct bins, which are characterized by decreasing similarity and increasing successful lure rejection rates. To be representative of varying degrees of similarity, we included equal amount of lure pairs with very high, medium, and low levels of similarity (labelled as Lure Bin 1, Lure Bin 3, Lure Bin 5, respectively in^13^). We selected 192 picture pairs of similar everyday object (i.e. 384 pictures altogether).

### Mnemonic similarity task

Task design is depicted in Fig. 1a. During encoding, we showed participants 128 pictures of everyday objects. Incidental encoding was applied. Participants were asked to decide whether the objects are used rather indoor or outdoor (response buttons were F and K, respectively). Following this, a set of 192 pictures were shown (there was no delay between the encoding and test phases). This set contained 64 target items (pictures seen in the encoding phase), 64 lure items (pictures, which were visually similar to, but not identical with one of the pictures seen in the encoding phase), and 64 foil items (previously unseen, new pictures). We asked participants to decide whether they saw the same picture as before, a similar picture, or a new picture. The response buttons were F (‘old’), H (‘similar’), and K (‘new’) on a standard keyboard.

In both the encoding and test phases, pictures were shown in the middle of the screen, on a white background. Each stimulus was shown for 2500 ms with an inter-stimulus interval of 2000 ms (see Fig. 1b).

Furthermore, because eye-movements can influence pupil size^77^, the pictures were presented in small format (under 5 visual degree) to minimize eye-movements. Besides, we also investigated gaze patterns during the period of stimulus presentation by plotting all fixations during this period (see Fig S3 for an example). As expected, most of the fixations were distributed in close proximity to each other – participants fixated mostly the center of the screen, where the objects were presented. Nevertheless, we could identify outlier fixations, for which either the x or thy y coordinate deviated by the more than three standard deviations from the mean of the given participants. We assumed that during such fixations participants looked away from the pictures – to avoid confounds in pupil size due to large eye-movements, trials with such outlier fixations were excluded from further analysis. On average, 4.13 percent of the trials (SD: 3.03) had to be excluded due to outlier fixations.

### Processing of pupil data

The diameter of the left eye’s pupil was estimated by the built-in algorithm of the eye-tracker, based on the amount of pixels occluded by the pupil on the video image of the eye-tracker. Data were preprocessed using a self-written MATLAB code. Because pupil size changes are relatively slow, the original data recorded on 1250 Hz were downsampled to 125 Hz. The vertical diameter of the pupil was measured.

Data filtering was conducted in several steps. First, using the built-in event-detection algorithm of the eye-tracker, data points identified as blinks were removed (ratio of removed data points: M = 6.40% SD = 6.68). To filter out noise associated with the movement of the eyelids before and after blinks, data from the 60 ms preceding and following blinks were also removed (ratio of removed data points: M = 3.67% SD = 2.60). Thereafter, all remaining data points with zero value xwere removed (ratio of removed data points: M = 0.33% SD = 0.73). After this, the data were separated to 192 short sequences of 10 secs. Each segment started at the presentation of an ISI, and consisted the period of picture presentation and also the subsequent ISI – this latter was included to assess the whole pupil response, which overlapped with the beginning of the next ISI after picture presentation (see Figs. 3, 4). Data points which were 2.5 standard deviations above the mean pupil size in a given data sequence were removed, and also data from the preceding and subsequent 60 ms (ratio of removed data points: M = 1.07% SD = 0.75).

Overall, 11.46% (SD = 8.01) of the data points were filtered. These missing data points were interpolated using linear interpolation. Trials with more than 30% of filtered data points were removed from analysis. On average, 9.36 (SD = 19.25) trials were removed – for one participant, an excessive amount of data sequences had to be removed (81 trials), thus his/her data was excluded from further analysis.

Thereafter, all data sequences were smoothed using a Savitzky-Golay filter (parameters: polynomial order:2, frame size:41) and standardized using the mean and standard deviation of that sequence.

### Sample size for the different analyses

Three participants had fewer than four observations for the condition new|target&lure. Because of this, they were excluded from all analysis involving this condition (i.e. analyses comparing correct and incorrect ‘new’ responses). Furthermore, in the control analysis without ‘new’ responses and foil items, five participants had less than four observations for the condition similar|target. Consequently, they were excluded from this analysis. In all other analysis, data from all participants were included.

### Pupil time course analysis

Time course analysis of pupil data was conducted with both stimulus- and response aligned data. For the analysis using stimulus-aligned data, we first segmented raw, preprocessed data into separate data samples of seven seconds representing each trial (from two seconds before until five seconds after stimulus presentation). Data in each segment was baseline corrected: we computed the mean pupil size for the 500 ms preceding stimulus presentation for each trial, and this mean value was subtracted from each data point of the given data segment. Thereafter, these trial-level data segments associated with different conditions were averaged for each participant and each condition separately. Finally, these participant-level condition-specific data samples were averaged separately for each condition to get the sample-level grand pupil size curve for each condition. These grand-average pupil size curves are presented on Figs. 3, 4.

Differences between the pupil size curves were done using paired-sample t-tests: for each data point, we selected from the participant-specific data samples those pupil size values, which belonged to the given time point and the to one of the to-be compared conditions (e.g. old|target or similar|lure). Then, using these data points of the two conditions, we conducted a paired-sample t-test to test whether there was significant difference between the two conditions in that given time-point. This procedure was repeated for each time point—differences between curves were interpreted where the paired-sample t-tests were significant for a consequitve period of at least 500 ms.

Response-aligned data was processed the same way, with two exceptions: first, we selected data segments for each trial by choosing data points between 3000 ms preceeding and 3000 ms following the key press on that given trial. Second, the way of baseline correction was different: First, the RT of the given trial was used to identify the onset of the stimuli in that given data segment. Then, the mean pupil size of the 500 ms preceeding this time point was calculated. Finally, this mean pupil size value was subtracted from each data point of the given data segment.

### Analysis of peak dilation

We first computed for each trial the peak dilation, that is the maximum value of pupil size. Because the examination of Figs. 3 and 4 suggest that the largest pupil size change can be observed parallel with the behavioral response, peak dilation was determined as the maximum pupil size value in the period of one second before and two seconds after the response. These peak PD values from each trial were separately averaged for each condition and each participant: For each participant, we computed peak PD for trials where he/she responded correctly with one of the three response types: that is we computed PDs for ‘old’ responses given to targets (old|target), for ‘similar’ responses given to lures (similar|lure), and for ‘new’ responses given to foils (new|foil). In a similar vein, for each participant, we also computed peak PD for trials in which he/she responded incorrectly with one of the three response types: that is, we computed ‘old responses given to lures and foils (old|lure&foil), for ‘similar’ responses given to targets and foils (similar|target&foil), and for ‘new’ responses given to targets and lures (new|target&lure). These average peak PD values were then used in repeated measures ANOVAs described in the results section. We used the Kolmogornov-Smirnof test to check whether the peak PD values for the different conditions are normally distributed or not. We found that all variables were normally distributed, thus the usage of parametric statistical methods was justified.

### Analysis of reaction times

Mean reaction time was calculated separately for responses to all relevant experimental conditions (e.g. old|target, similar|target&foil). Because we wanted to examine the link between PD and RT, only trials were included for which we also had valid pupil size data (i.e. trials excluded from pupillometric analysis due to poor data quality or outlier fixations were also excluded from RT analysis).

#### Linear mixed modelling

We used the lme4 package in R^78^ to conduct trail-level linear-mixed model analysis. We first built a base model which predicted trial-level PDs by participant-specific differences and by the illuminance of the picture presented on the specific trial. The random effect structure of this model was random intercept for both illuminance and participant. In the second step, we added a dichotomous variable coding the to-be-comparable conditions, as fixed effect. Then, we investigated whether model with the fixed effect significantly increased model fit, as compared to the base model – significant improvement was taken as an evidence for a significant fixed effect.

#### Luminance control of stimuli

Pupil size is sensitive to changes in stimulus luminance^60–64^: due to a complex interaction of the sympathetic and the parasympathetic system, pupil size decreases when stimulus luminance increases (pupillary light reflex), and decreases when stimulus luminance increases (darkness reflex). Because of this, one of the most important methodological aspect of pupillometric studies is to control stimulus luminance differences. This is often achieved by using isoluminant stimuli. However, we did not want to alter the stimuli, as they were validated as effective lures in their original, colored form in Stark et al.^13^. Furthermore, even the presentation of isoluminant stimuli can lead to perceptually driven pupil size changes^63,79,80^.

Because of this, we did not try to eliminate noise from perceptual sources, but aimed to hold this type of noise random and opted to use the original, colored pictures and to counterbalance them between conditions. To this aim, we designed our task in a way that each picture was assigned to each condition equally often- with this manipulation, luminance differences of pictures presented in the different conditions was equated. Specifically, we selected 192 picture pairs from the database used in several previous studies (e.g.^13,17,19^). Each picture depicted an everyday object, and each picture pair depicted similar objects (i.e. they were each other’s lures). We created three stimulus groups of 64 picture pairs – in each stimulus group, the amount of pictures from the three lure bins was equated (lure bin 1, 3 and 5). Then, we built six different stimulus sets, with all possible combination of the assignment of stimulus group to experimental condition (target, lure, foil). Each participant was administered randomly one of the stimulus sets, and so the luminance features of different pictures randomly influenced the average luminance of the lure, target and foil conditions.

Furthermore, we also took in account the fact that light reflex and the darkness reflex have a differential time course^64^. Thus, additional variability in the pupil signal might be expected if some of the presented stimulus activates the light reflex, whereas other pictures engage the darkness reflex. This can be the case, if some of the pictures are lighter, whereas other are darker than the background color of the screen presented before the picture. To avoid this, we used a white screen as background color during both the inter-stimulus interval (ISI) and during stimulus presentation (see Fig. 1b). Because a plain white surface has higher luminance than any colored picture, this stimulus outlay ensured that the luminance of the fixated area decreased after presentation of the MST items. With this manipulation, we granted that luminance related pupil size changes are always mediated by the darkness reflex, whereas the light reflex is not involved.

Nonetheless, whereas this randomization assures that luminance features of the pictures randomly influence pupil responses in different conditions, it is not fully able to eliminate luminance confounds when comparing pupil responses accompanying responses to groups of stimuli in the same condition. For example, when comparing pupil dilation to lure items associated with ‘old’ versus ‘similar’ responses, the picture luminance levels cannot be a priori randomized, as they are dependent on the response of the participant. To control for spurious effects arising from this, we investigated whether our results might be confounded by luminance differences between the stimuli present for correct and incorrect trials.

Due to the special features of our particular design and the applied stimuli, instead of stimulus luminance we monitored the illuminance at the location where the participants’ head are placed. For explanation let us consider the difference between the two mentioned quantities. Luminance, at the observer’s eye, is the amount of light incident from a given direction to unit projected area within unit solid angle. In contrast, illuminance is the total amount of light per unit area incoming from the entire half space. According to former measurements, the accommodated pupil size is determined by both the luminance produced by the fixated object, and the solid angle which it subtends at the eye (for a review, see^81^). These authors found that pupil diameter changes are driven by the product of luminance and solid angle, which is the illuminance (at least for small angles). In our design, test objects were presented on a white screen. This occupied a large portion of the participant’s visual field, and so in itself it was a significant source of illumination strongly affecting the pupil size. The pixels of the test objects covered only a small area on the screen. Since the individual pixels are observed from different directions by the eye, their actual value specifies the luminance in that given direction. Turning one pixel black results in zero luminance, however, it causes little change in pupil diameter. This is in correspondence with the fact that switching one pixel black hardly changes the illuminance, which varies depending on the number of non-white pixels and their luminance value. Because of this, the level of illuminance can be better used to characterize stimulus changes than luminance differences. Importantly, background illumination in the room was constant during the task, thus any differences in illumination measured for the subsequent test pictures were due to differences in stimulus illuminance.

To measure illuminance, a digital light meter (type: SBS-LM-200C) was fixed at the top of the chin-rest of the eye-tracker, approximately at the position where the eye’s of the participants are located during the experiment. The mean illuminance measured during presenting of the 384 pictures was 42.95 lx (SD = 0.49, range: 41.2–43.9). During the white background screen, illuminance was 44 lx.

## Supplementary information

Supplementary information 1.

Supplementary information 2.

## Data Availability

All data generated or analysed during this study are included in this published article (and its Supplementary Information files).
