# Supplementary material for: Pupil size changes signal hippocampus-related memory functions
Source: Sci Rep. 2020 Oct 2;10:16393. doi: 10.1038/s41598-020-73374-9 (PMC7532445; doi:10.1038/s41598-020-73374-9)
Supplement: Supplementary file 2 — Supplementary information 2. [file 41598_2020_73374_MOESM2_ESM.pdf]

Pupil size changes signal hippocampus-related memory functions

Péter Pajkossy1,2 *, Ágnes Szőllősi1,2, Mihály Racsmány1,2

1 Institute of Cognitive Neuroscience and Psychology, Research Centre for Natural Sciences, Hungary

2 Department of Cognitive Science, Budapest University of Technology and Economics, Budapest, Hungary

Supplementary material

A) Illuminance differences between conditions

As can be seen on Supplementary Fig. S1, there is a weak negative correlation between illuminance and PD, which suggest that lower illuminance following the white screen presented during the ISI evokes a larger darkness reflex and this leads to larger pupil response. There was an increase in illuminance with increasing lure similarity (Lure bin 1 -strong similarity: M=43.06, SD=0.43; Lure bin 3- medium similarity: M = 42.95, SD=0.53; Lure bin 5 - weak similarity: M=42.85, SD=0.49). The difference between lure bins was significant, $F(2,383) = 6.19$, $p = .002$. Using paired samples t-test, we showed that there is a significant difference in luminance between strong and weak similarity, $t(254)=3.69$, $p<.001$, $d= 0.45$, whereas the other two comparisons failed to reach significance (medium vs. strong similarity: $t(254)=1.89$, $p =.06$, $d= 0.23$; medium vs. weak similarity: $t(254)=1.55$, $p =.12$, $d= 0.19$)

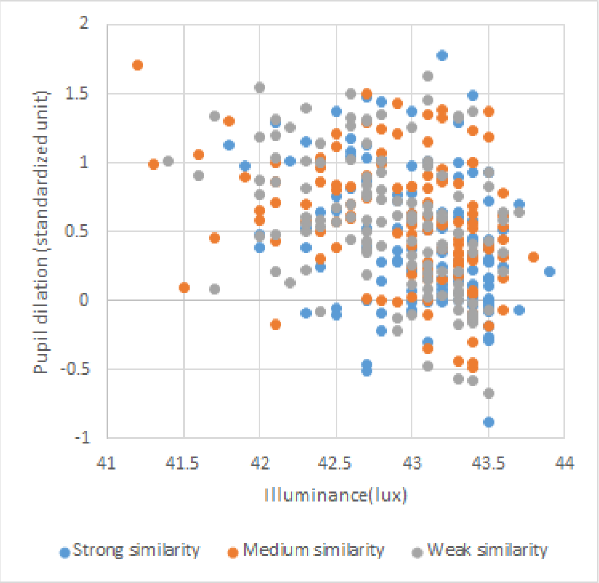

S1. Scatterplot between evoked pupil dilation and measured illumination for each MST picture.

To check, whether this link between illuminance and PD influences our results, we calculated for each participant the average illuminance during the pictures presented in different experiential conditions. (see Fig. 1e for mean illuminance in the different conditions). Illuminance of pictures presented during correct responses of participants were not different from each other, F(2,40)=1.75, p = .40, $\eta_p^2 = .05$. Contrast analysis revealed that there is no significant difference between either correct 'similar' and 'old' responses, F(1,20)=2.81, p = .11, $\eta_p^2 = .13$, or between correct 'old' and 'new' responses, F(1,20)=2.69, p = .12, $\eta_p^2 = .12$, or between correct 'new' or 'similar' responses, F(1,20)=0.18, p = .67, $\eta_p^2 = .01$.

Furthermore, the two-way repeated measures ANOVA with judgement veridicality (correct vs. incorrect) and response type ('old' vs. 'similar'), as within-subject factors, and illuminance, as dependent variable revealed a near significant effect of judgement veridicality, F(1,20)=4.31, p = .051, $\eta_p^2 = .18$, whereas the effect of response type and the interaction of the factors was not significant, F(1,20)=2.37, p = .14, $\eta_p^2 = .11$ and F(1,20)=0.01, p = .98, $\eta_p^2 = .01$, respectively. There was a significant difference between the illuminance of correct and incorrect 'old' responses, t(20)=3.07, p=.006, d=57, whereas no significant difference was found for either correct and incorrect 'similar' responses, t(20)=1.28, p=.22, d=0.31, or 'new' responses, t(17)=0.533, p=.60, d=0.14.

B) Peak dilation analysis using stimulus-aligned data

Peak pupil dilation values for each trial were computed by choosing the maximum pupil size value for each trial in the time period starting from stimulus onset and lasting until 4000 msec after stimulus onset. Peak pupil dilation values were averaged for each participant and for each condition the same way, as described in the results and method section for the peak-dilation analysis with response aligned data (see Fig S2 for average values). These peak pupil dilation values were then used to conduct the same analyses, which were done using response-aligned data.

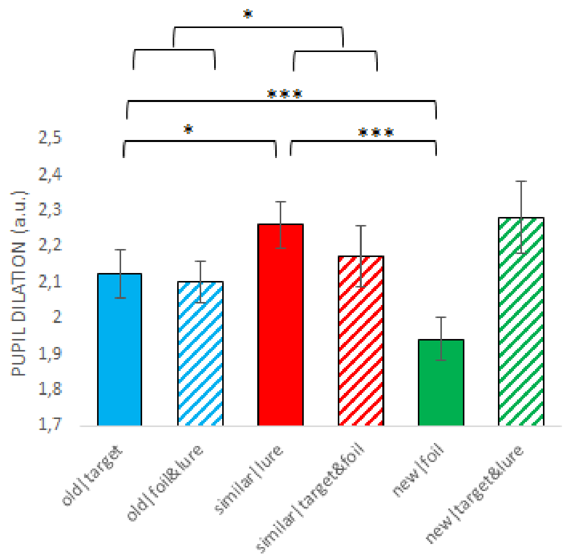

Figure 2. Differences between the experimental conditions in stimulus-aligned pupil dilation

Mean pupil dilation for correct and incorrect 'old', 'similar' and 'new' responses. Significance is indicated only for comparisons which were reported in the statistical analyses. Due to participant exclusion, the bar representing incorrect 'new' responses to foils represents data from only 18 participants, whereas the other bars represent data from all participants. Because of this, significance between the difference for correct and incorrect 'new' responses are not indicated with asterisk on the graph.

Note: *: p <.05; **: p <.01; ***: p<.001; Error bars represent the standard error of the mean.

First, we investigated whether correct responses are associated with different PDs using a repeated measures ANOVA with response type as within-subject variable ('old', 'new', similar'). We found a significant main effect, $F(2,40)=18.65$, $p < .001$, $\eta_p^2 = .48$. Contrast analyses showed that all three response types differed from each other (old|target vs. similar|lure: $F(1,20)=6.05$, $p = .02$, $\eta_p^2 = .23$;

old|target vs. new|foil: $F(1,20)=21.66$, $p < .001$, $\eta_p^2 = .52$; similar|target vs. new|foil: $F(1,20)=28.45$, $p < .001$ .31, $\eta_p^2 = .59$).

Second, we investigated the role of judgement veridicality by using a 2x2 repeated measures ANOVA with response type (‘old’ vs. ‘similar’) and judgement veridicality (‘correct’ vs. ‘incorrect’), as within subject factor. We found a significant main effect of response type, $F(1,20)=5.55$, $p = .03$, $\eta_p^2 = .22$, whereas the effect of judgement veridicality and the interaction of the factors was not significant ($F(1,20)=1.63$, $p = .22$, $\eta_p^2 = .08$ and $F(1,20)=0.50$, $p = .54$, $\eta_p^2 = .02$, respectively).

Finally, we compared correct and incorrect ‘old’ and ‘new’ responses, respectively. We found no significant differences between the old|target and the old|foil&lure condition, $t(2) = 0.38$, $p = .70$, $d=0.07$, whereas there was a significant difference between new|foil and new|target&lure condition, $t(2) = 3.72$, $p = .002$, $d=0.85$.

C) Example fixation map of a participant during stimulus presentation

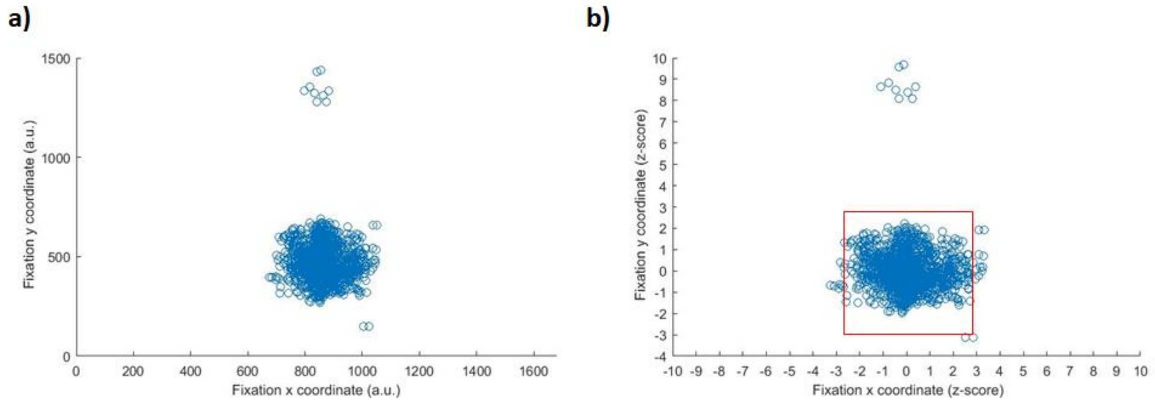

Fig S3. The selection of outlier fixation for one specific participant.
As can be seen on Fig S3a, most fixations were distributed relatively close to each other. After standardizing the x and y coordinates, all trials were excluded, where at least one fixation was found with a z-score above 3 or below -3 (i.e. all fixations outside the red rectangle)
